# Effect of N-Carbamylglutamate Supplementation in Late Pregnancy on Nutrient-Restricted Twin-Bearing Ewes on the Pre-Lambing Maternal Metabolome, Colostrum Quality and Lamb Birth Weight

**DOI:** 10.3390/ani15202998

**Published:** 2025-10-16

**Authors:** Francisco Sales, Susan McCoard, Pablo Alarcón, Camila Sandoval, Claudia Silva, Carolina Rojas, Víctor H. Parraguez

**Affiliations:** 1 Department of Animal Science, INIA Kampenaike, Punta Arenas 6212707, Chile; fsales@inia.cl (F.S.); csandovalt@udec.cl (C.S.); 2 AgResearch Grasslands, Private Bag 11008, Palmerston North 4442, New Zealand; sue.mccoard@agresearch.co.nz; 3 Faculty of Veterinary Sciences, Universidad Austral de Chile, Valdivia 5090000, Chile; pabloalarcon.u@gmail.com; 4 Department of Animal Science, University of Concepción, Chillán 3800708, Chile; 5 Faculty of Veterinary Sciences, University of Chile, Santiago 8820808, Chile; claudia.silva.m@ug.uchile.cl (C.S.); caro.rt1302@gmail.com (C.R.); 6 Faculty of Agrarian Sciences, University of Chile, Santiago 8820808, Chile

**Keywords:** arginine, maternal-fetal nutrition, colostrum immunoglobulins, multiple pregnancy, sheep production systems, metabolomics

## Abstract

**Simple Summary:**

Lamb mortality around birth, especially in twin lambs, is of major concern for producers. Under natural nutrient restriction, arginine, a conditionally essential amino acid for the fetus, is known to improve fetal growth and colostrum quality in restricted ewes. However, its use is limited in ruminants due to its high cost and degradation in the rumen. N-carbamylglutamate (NCG) can stimulate the body’s own arginine production and may serve as an alternative. This study evaluated the effects of NCG supplementation during late pregnancy in undernourished twin-bearing ewes. Ewes received 60 mg/kg body weight of NCG daily from day 100 of gestation until lambing. Compared to unsupplemented controls, NCG did not improve ewe body condition, placental traits, or lamb birth weight. However, NCG supplementation resulted in changes in maternal blood metabolites, including higher plasma urea and altered amino acid profiles. Importantly, colostrum from NCG-treated ewes had significantly higher protein and IgG content. While NCG did not reverse the negative effects of undernutrition on fetal growth, it did improve colostrum quality and immunoglobulin content, which may enhance immunity in lambs and contribute to reducing lamb mortality after birth, a key constraint in extensive sheep production systems.

**Abstract:**

Arginine supplementation improves fetal growth and colostrum composition in nutrient-restricted ewes, but its high cost and ruminal degradation limit its practical use. N-carbamylglutamate (NCG), which stimulates endogenous arginine synthesis, and is not degraded in rumen, may be a viable alternative. This study evaluated the effects of oral NCG supplementation (60 mg/kg BW/day from day 100 of gestation to term) on undernourished twin-bearing ewes (~50% NRC requirements; NCG *n* = 20, Control *n* = 21). Maternal body weight (BW), body condition score (BCS), blood metabolites, placental traits, lamb body measurements, and colostrum composition were assessed. BW increased and BCS decreased over time (*p* < 0.0001), with no treatment effect. Lamb and placental traits were similar between groups. NCG supplementation resulted in a 15% higher plasma urea concentration (*p* < 0.03) and altered 21 serum metabolites, with reduced levels of valine, leucine, isoleucine, glycine, proline, and phosphate, and increased serine, ethanolamine, urea, and 2-hydroxyhexanoic acid concentration compared to CON animals. Colostrum from NCG ewes had a 21% higher protein (*p* < 0.04) and a 16% higher IgG content (*p* < 0.03) compared to CON animals. Although NCG did not mitigate the negative effects of maternal undernutrition on fetal traits, it influenced maternal metabolism and improved colostrum quality.

## 1. Introduction

The sheep industry in Patagonia is based on extensive grazing systems, relying on natural grasslands as the main feed source [1]. With a harsh environment and reduced pasture and nutrient allowance [2], undernutrition during gestation is a key productive constraint [3]. A reduction in maternal body weight and body condition score commonly occurs during the last third of gestation, with body condition losses reaching up to 40%, as extensive Patagonian prairie systems are unable to meet pregnancy nutrient requirements [4]. This is more severe in twin gestations as nutrient requirements are 40 to 50% higher in multiple gestations compared to singletons [5,6]. The resulting effect is an increase in lamb mortality around birth, especially in twins [7]. This is in agreement with previous reports from other latitudes, with twin-born lamb mortality being ~35% of the total reproductive wastage [8], and the majority of lamb losses occurring during the first three days postpartum [9].

This is of major economic and animal welfare concern for producers [10]. Although several factors have been described as contributing to lamb mortality after birth, lamb birth weight and litter size are considered the main factors [11]. In addition, low maternal nutrient intake compromises colostrum production and composition, including IgG concentration, impacting on newborn lamb passive immunity, health and survival [12]. Therefore, several studies have attempted to elucidate potential maternal interventions to improve fetal growth and/or colostrum quality, especially in nutrient-restricted multiple-bearing ewes [5,13].

One promising approach for intervention is maternal supplementation with L-Arginine (Arg), a versatile and conditionally essential amino acid for the fetus as its production may not be sufficient to cover the demand for placental and fetal growth, especially during periods of compromised nutrition [14,15]. Arg supplementation has been evaluated in maternal nutrient restriction models in sheep, resulting in a 21% increase in fetal growth [16] and a 23% of survival rate [17] in multiple gestations. In addition, twin-bearing ewes supplemented with Arg during late pregnancy improves colostrum protein content (~10%) [18]. Similarly, supplementation to sows during late gestation increases IgG content in the colostrum [19,20], which makes Arg an interesting candidate for targeting interventions in nutrient-restricted environments. However, despite the benefits of maternal supplementation with Arg, use in commercial farming practice is limited as it is degraded in the rumen [21]; therefore, its oral use is inefficient and expensive [22], even in the rumen-protected formulations [23].

During the last decade, there has been an increased interest in N-carbamylglutamate (NCG), a biologically stable isotype of N-acetylglutamate, an activator of carbamoyl-phosphate synthetase-1, a key enzyme that promotes the endogenous synthesis of Arg, via activation of the of urea cycle [24]. In contrast to Arg, NCG is not degraded in rumen [21,25], and, in addition to its use by rumen epithelial and duodenal cells for urea synthesis [26], NCG completely enters the systemic circulation in adults [27]. Furthermore, NCG is substantially cheaper than Arg [28], and its safety for animal use has been demonstrated [24]. Studies have shown that maternal supplementation with NCG in an undernourished sheep model may improve fetal growth. However, there are contrasting results when dietary management and timing of supplementation differ. In nutrient-restricted twin-bearing ewes (50% National Research Council, NRC), supplementation with 2.5 g/d of NCG from day 35 to 110 days of gestation (dg) increased fetal weight at 110 days of gestation by 19% compared to unsupplemented controls [29]. A metabolomic study suggest the NCG effect, in addition to promoting the urea cycle, could potentially be driven via altered protein and lipid metabolism and oxidative stress metabolic pathways [30]. In contrast, in severely undernourished twin-bearing ewes (~30% of NRC requirements) supplemented with 60 mg/kg of NCG (3.6 g/d) from 100 to 140 dg, there was no effect on fetal weight at 140 dg [31]. Therefore, the impact of maternal supplementation with NCG on fetal growth may be conditional on the level and timing of supplementation and/or the degree of nutrient restriction. In addition, prior studies suggest that NCG supplementation in dairy cattle may improve milk production [28,29,30]; however, to date, there are no reports on the effect of NCG on ruminant colostrum composition.

The aim of this study was to evaluate the effect of oral NCG supplementation from 100 days of gestation to term in undernourished twin-bearing ewes (~50% NRC requirements). Specifically, we aimed to assess the effect of NCG on maternal and lamb performance, the effect on colostrum composition and to investigate the impact on the maternal metabolome. We hypothesize that NCG supplementation in severely nutrient-restricted twin-bearing ewes during the last third of gestation will improve lamb birthweight and colostrum composition by modulating key maternal metabolic pathways.

## 2. Materials and Methods

### 2.1. Ethics Statement

This study was performed in agreement with the *Guide for Care and Use of Laboratory Animals* (Eighth Edition, National Research Council, National Institutes of Health, USA). The protocol was approved by the Bioethics Review Committee of the Instituto de Investigaciones Agropecuarias (INIA, Ministry of Agriculture, protocols No. 07-2022 and 12-2023).

### 2.2. Animals and Experimental Procedure

This study was conducted at the INIA-Kampenaike Research Farm, 60 km north of Punta Arenas, Chile (Chilean Patagonia, Lat. 52°41′ S Long. 70°54′ W). A total of 500 Corriedale ewes (4–6 years old) from a commercial flock were synchronized using a controlled internal drug release (CIDR G^®^, Zoetis, Santiago, Chile) device for 12 days, followed by 300 IU eCG (Novormon, Syntex, Argentine) i.m. injection. Mating was carried out via laparoscopic artificial insemination, using semen from a single Corriedale ram, in order to standardize the paternal effect. All animals were maintained under grazing conditions in the same paddock according to farm management protocols and with a stocking rate of 0.9 animals/ha, receiving no supplementation in order to maintain the normal sheep-rearing conditions encountered in Patagonia. Transabdominal ultrasound pregnancy examination was done 80 days after insemination (Oviscan, BCF, Technology Ltd., Livingston EH54 8TE, Scotland, UK) to confirm pregnancy status, litter size and estimated fetal age. Forty-two twin-bearing ewes, with similar gestational age were selected and included in the study. However, one animal had to be excluded later due to health problems, leaving 41 individuals. Body weight (BW) and body condition score (BCS, 1 to 5 scale [32]) were 57.6 ± 6.3 kg and 1.9 ± 0.5, respectively, at the start of the experiment.

At 100 days of gestation (dg), ewes were randomly assigned into two groups. The first group, was orally dosed once daily with 60 mg/kg BW NCG (Inner Mongolia Tianyi Sci. & Tech. Co., Ltd., Ulanqab, Inner Mongolia, China) in a water carrier from 100 dg to lambing (NCG; *n* = 20). This dose has been previously shown to increase plasma NCG concentration in pregnant sheep [31]. NCG doses were prepared daily according to individual BW at 100 dg and adjusted every 15 days according to changes in BW. The second group was the unsupplemented control (CON; *n* = 21), receiving only the water carrier, to emulate the handling procedure of NCG animals. The ewes were managed in individual pens from day 100 until lambing, receiving an individually determined daily ration covering 50% of their protein and energy requirements [6], with alfalfa hay (60% of the diet containing 85.55% DM; 12.12% CP; 2.10 Mcal/kg ME and 55.92% D-value) and natural pasture hay (40% of the diet; 84.0% DM; 6.85% CP; 2.13 Mcal/kg ME and 56.8% D-value) to mimic undernutrition levels used in a previous NCG study [33]. Due to the low body condition score at the start of the experiment and the imposed nutrient restriction, animals were observed daily according to the Morton and Griffiths [34] protocol to assess appearance (e.g., wool loss, abnormal posture, diarrhea, lameness), feed intake (via daily refusal assessment) and water intake, body weight and condition, behavior, and clinical signs. An individual score above 15 on this scale was used as a criterion to withdraw the animal from the experiment. Food intake was measured daily via refusals; however, animals consumed 100% of the diet on every single day. Animals received water ad libitum. Maternal BW and BCS were recorded every 15 days, from 100 dg until lambing. Body weight, morphometric measurements (body length, biparietal diameter, thorax diameter, front and hind leg length), and rectal temperature of the lambs were recorded at birth. In addition, gestation length was also calculated. Placenta expulsion after lambing was monitored to prevent maternal consumption. The fetal cotyledons and associated membranes were collected after expulsion, and their total weight, total cotyledon number, and total cotyledon weight were obtained, as previously described [35]. As placentas were collected after delivery, it was not possible to determine placental characteristics per lamb. Data are therefore reported considering both placentas together.

### 2.3. Assessment of Maternal Blood Metabolites

Maternal blood samples were obtained around 10 a.m. at 130 dg, 145 dg, and 30 min after parturition by jugular venipuncture, using a vacuum serum tube (BD Vacutainer, Franklin Lakes, NJ, USA). Samples were maintained at ambient temperature for 30 min, and serum was separated after centrifugation at 3000× *g* for 10 min at 4 °C, and stored at −80 °C. Concentrations of β-hydroxybutyrate (enzymatic method; Randox^®^, Crumlin, UK, kit no. RB 1107), urea (enzymatic-colorimetric method; Human^®^, Wiesbaden, Germany, kit no. 10505), total protein (colorimetric test; Human^®^, Wiesbaden, Germany, kit no. 157004), and albumin (colorimetric test; Human^®^, Wiesbaden, Germany, kit no. 10570) were determined by a commercial veterinary laboratory (Survet, Osorno, Chile) following the manufacturer’s instructions.

#### Sample Preparation for Metabolite Extraction

Metabolites were extracted from 16 maternal serum samples (8 CON and 8 NCG) obtained prepartum (145 dg), following the protocols previously described [36,37], with minor modifications. Briefly, 100 µL of ovine serum was extracted with 1 mL of a cold acetonitrile–isopropanol–water (3:3:2; GC/MS grade) mixture, containing 1 mM adonitol (#A5502, Sigma-Aldrich, MO, USA) as an internal standard. The samples were vortexed for 30 s, placed on ice for 5 min, and centrifuged at 14,000× *g* for 5 min at 4 °C. After centrifugation, 450 µL of the supernatant was evaporated to dryness using a SpeedVac™ Concentrator (Savant^®^ SPD131DDA, Thermo Fisher Scientific, MA, USA). The residue was washed and dried twice with 450 µL of 50% *v*/*v* acetonitrile in water (GC/MS grade) using the SpeedVac™.

For derivatization, 5 µL of a fatty acid methyl ester (FAME) standard mixture (C8–C30; #400505, Fiehn GC/MS Metabolomics Standards Kit, Agilent Technologies) and 10 µL of methoxyamine hydrochloride/pyridine solution (20 mg/mL; #226904, Sigma-Aldrich, and #107463, Merck, Darmstadt, Germany) were added to the dried samples. The samples were incubated at 30 °C for 90 min in a benchtop orbital shaker. Subsequently, 90 µL of derivatization reagent [N-methyl-N-(trimethylsilyl)-trifluoroacetamide (MSTFA) with 1% trimethylchlorosilane (TMCS); #69478, Merck] was added, and the samples were incubated at 37 °C for 30 min. The derivatized samples were transferred to 250 µL glass vial inserts within 1.5 mL glass vials with screw caps for GC-MS analysis.

Derivatized samples were injected into an Agilent 7890B GC system with an electron impact (EI) ionization mode 5977A (MSD, Agilent Technologies, Palo Alto, CA, USA). A 1 µL aliquot of each derivatized sample was injected in splitless mode onto a 30 m × 0.25 mm × 0.25 µm DB-5 column (Agilent Technologies). The injector port temperature was maintained at 250 °C, and the helium carrier gas flow rate was set to 1 mL/min. The oven temperature was initially set at 60 °C and increased by 10 °C/min until reaching 325 °C, with a total run time of 37.5 min. A 5.9-min solvent delay was applied, and full spectra (50–600 *m*/*z*; 1.7 scans/s) were acquired at a digital scan rate of 20 Hz. The MS ion source and quadrupole temperatures were set to 250 °C and 150 °C, respectively. All samples were analyzed within 24 h of derivatization. Retention times for calculating the Fiehn retention index (RI) were determined using a C8–C30 FAME standard mixture (#400505, Fiehn GC/MS Metabolomics Standards Kit, Agilent Technologies).

Raw MS data in the ‘.D’ format were converted to ‘.Abf’ format using Reifycs Abf Converter 4.0.0 software (RIKEN Center for Sustainable Resource Science, Yokohama, Japan) before data pretreatment. Peak detection, deconvolution, and alignment were performed using MS-DIAL 4.70 software. After deconvolution, the purified mass spectra of trimethylsilylated metabolites were matched against Fiehn’s mass spectral library. Matches were ranked by retention index, mass spectral similarity, and alignment with batch-processed experimental data. Identification criteria included a 2000 RI tolerance, 70% EI similarity cut-off, 70% identification score cut-off, 0.5 Da *m*/*z* tolerance, and 0.5-min retention time tolerance.

### 2.4. Colostrum Analysis

Colostrum (50 mL) was collected 15–30 min after the second lamb was delivered, from both sides of the udder, before the lambs began suckling. Samples were heated to 38 °C and homogenized on a shaker for 3 min. They were then diluted in deionized distilled water (1:1 *v*/*v*), homogenized, and evaluated at 23 °C. Fat, non-fat solids, density, and protein contents were determined using an Ekomilk^®^ analyzer (Milkana KAM98-2A, Bulteh 2000 Ltd., Stara Zagora, Bulgaria). The IgG concentration was measured in colostrum samples by radial immunodiffusion (RID), as previously described [38].

### 2.5. Statistical Analysis

The effect of NCG supplementation on ewe BW, BCS, and blood metabolites was analyzed using Restricted Maximum Likelihood (REML) analysis in R, using the lme4 R package (R version 4.3.0) with time (dg) and treatment (and their interaction) as fixed effects. Lamb BW, morphometric measurements, and rectal temperature were evaluated considering treatment, sex of the fetus, and their interaction as fixed effects, with ewe as a random effect to account for twin pregnancies. Lamb morphometric measurements were adjusted for lamb weight as a covariate. Placental data, gestation length, and colostrum variables were analyzed using REML analysis in R, using the lme4 R package, with treatment as a fixed effect and ewe as the random effect. Treatment differences were considered significant if *p* ≤ 0.05.

For metabolomic data analysis, multivariate statistical analyses were conducted using MetaboAnalyst v4.0 (http://www.metaboanalyst.ca (accessed between 1 July 2024 and 29 September 2025); Xia Lab, McGill University, Canada), as described in previous studies [36,37]. Metabolite concentrations were normalized using adonitol as an internal standard. To achieve a Gaussian distribution, logarithmic transformation and auto-scaling were applied before statistical analysis. Principal Component Analysis (PCA) was conducted to visualize overall differences in the serum metabolome between NCG-supplemented and control ewes and to reduce data dimensionality. The proportion of variance explained by each principal component was used to evaluate model adequacy. Statistical significance of group separation was assessed using permutational multivariate analysis of variance (PERMANOVA, 999 permutations), which yielded F = 31.231, R^2^ = 0.69048, and *p* = 0.001. Heat maps were generated using Euclidean distance measures and Ward’s clustering algorithm. Metabolites with significantly different levels (*p* ≤ 0.05, Mann–Whitney test) were included in pathway topology analyses. *Ovis aries* (sheep) was used as the model organism for pathway analysis, employing the *Ovis aries* pathway library. Overrepresentation analysis was performed using a hypergeometric test. Potential metabolomic pathways were identified with the Kyoto Encyclopedia of Genes and Genomes (KEGG; http://www.genome.jp/kegg (accessed between 1 July 2024 and 29 September 2025)) and the Bovine Metabolome Database (http://www.cowmetdb.ca (accessed between 1 July 2024 and 29 September 2025)).

## 3. Results

### 3.1. Maternal Body Weight and BCS

**Figure 1:**
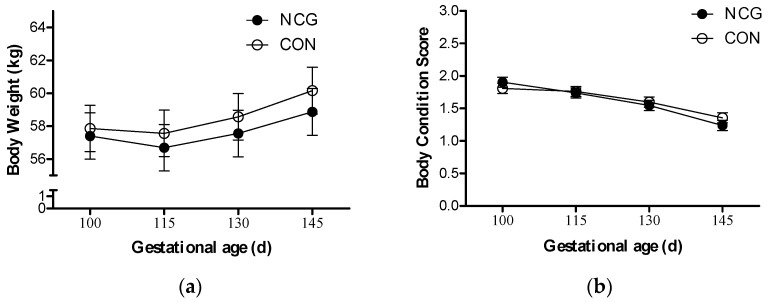
Body weight (**a**) and body condition score (**b**) variation from day 100 to 145 of gestation of NCG-supplemented (NCG) and unsupplemented control (CON) undernourished ewes.

At the beginning of the study, BW and BCS were similar between groups. As gestation advanced, ewe BW increased on average in both groups by 4% (*p* < 0.0001) (Figure 1a), and BCS decreased by 28% (*p* < 0.0001) (Figure 1b), but there was no effect of treatment or a treatment–time interaction (*p* > 0.05).

### 3.2. Lamb Body Weight and Biometric Variables

No differences were observed in lamb birth weight between groups (*p* > 0.05). Similarly, lamb biometric dimensions were similar between CON and NCG groups. A sex effect was observed for hind limb length, with males having 3% longer hind limbs than females (36.1 ± 0.43 vs. 35.1 ± 0.38 cm, *p* = 0.04). A similar sex effect was observed for forelimb length, with males having 3% longer forelimbs than females (30.4 ± 0.32 vs. 29.5 ± 0.29 cm, *p* = 0.02). No treatment effect was observed in gestation length or lamb rectal temperature 30 min postpartum (*p* > 0.05). Total weight of fetal membranes and cotyledons, cotyledon weight, and cotyledon number did not differ between groups (*p* > 0.05) (Table 1).

### 3.3. Maternal Blood Metabolites

There was an effect of treatment on maternal blood urea concentration, with NCG ewes having a 26.5% higher concentration than CON ewes in late gestation (*p* = 0.03) (Table 2). No differences were observed for β-hydroxybutyrate, total protein, or albumin between groups (*p* > 0.05).

### 3.4. Colostrum Composition and IgG Concentration

Colostrum protein content was 21% higher in NCG compared to CON ewes (*p* = 0.04); however, no differences were observed for fat, non-fat solids or density (*p* > 0.05) between groups (Table 3). Colostrum IgG concentration was 16% higher in ewes from NCG group, compared to CON ewes (*p* = 0.03).

### 3.5. Maternal Serum Metabolome at 145 dg

#### 3.5.1. Principal Component Analysis (PCA)

**Figure 2:**
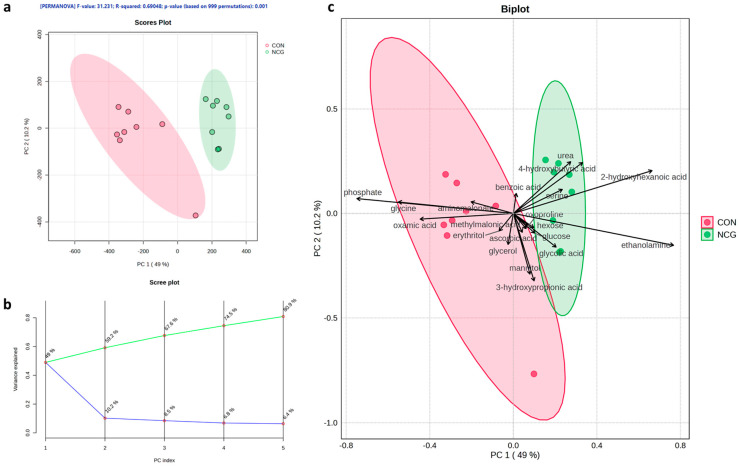
Principal Component Analysis (PCA) of maternal serum metabolomic profiles in undernourished twin-bearing ewes supplemented with N-carbamylglutamate (NCG) or unsupplemented controls (CON). (**a**) The data points for each group are clustered, with ellipses representing the 95% confidence intervals around each group’s centroid. Colors correspond to experimental groups: CON (pink) and NCG (green). The *x*-axis (Component 1) and *y*-axis (Component 2) explain 10.2% and 49.0% of the total variance in the data, respectively. The reliability of the model is supported by a PERMANOVA test (F-value = 31.231, R^2^ = 0.69048, *p* = 0.001, based on 999 permutations). (**b**) Scree plot of explained variance for the first five principal components (blue line), indicating that cumulative variance reaches 80.5% (green line). (**c**) PCA biplot displaying the main metabolites contributing to separation between groups, with colors corresponding to CON (pink) and NCG (green).

After deconvolution and alignment, a total of 186 metabolites based on *m*/*z* ratio were identified, including organic acids (*n* = 70), amino acids and derivatives (*n* = 31), sugar alcohols (*n* = 20), miscellaneous compounds (*n* = 23), amines and other nitrogen-containing compounds (*n* = 14), sugars (*n* = 11), lipids and fatty acids (*n* = 4), phosphates and cofactors (*n* = 4), and nucleotides and bases (*n* = 8). Principal Component Analysis (PCA) of the maternal serum metabolome revealed a clear separation between CON and NCG groups (Figure 2a). The first two principal components explained 59.2% of the total variance (PC1 = 48.8%, PC2 = 10.1%), and their combined effect was statistically significant (PERMANOVA, F = 31.23, R^2^ = 0.69, *p* = 0.001). To evaluate the robustness of the model, we further examined the variance explained by additional components. The scree plot showed that the first five PCs together accounted for 80.5% of the cumulative variance (Figure 2b), supporting the adequacy of the PCA model. The biplot analysis (Figure 2c) indicated that branched-chain amino acids (valine, leucine, isoleucine), glycine, and proline had the highest loadings on PC1 and were more abundant in CON ewes. In contrast, metabolites such as serine, ethanolamine, and 2-hydroxyhexanoic acid contributed mainly to PC2 and were elevated in NCG ewes. These results align with the univariate analysis, confirming that NCG supplementation modified specific amino acid and nitrogen metabolism pathways. Detailed eigenvalues of the first five principal components and the loading scores of the top contributing metabolites for PC1 to PC5 are provided in Supplementary Table S2.

**Figure 3:**
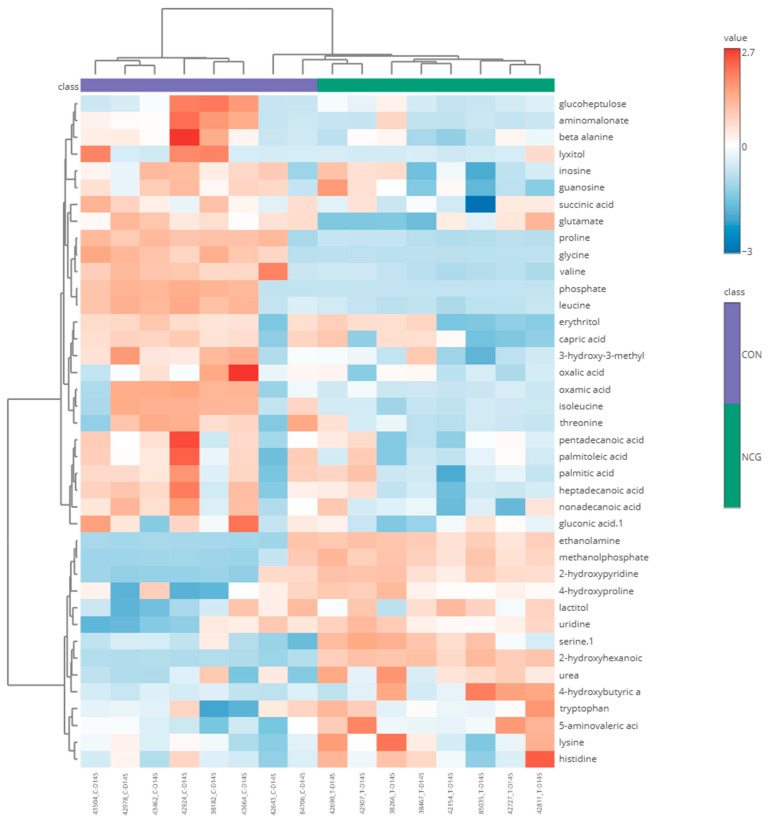
Clustering of 40 metabolites identified, driven by characteristic metabolic patterns, shown as heatmap (distance measure using Euclidean, and clustering algorithm using ward.D). The heatmap illustrates the relative abundance of blood metabolites in sheep following N-carbamylglutamate (NCG) treatment compared to the control (CON) group. Hierarchical clustering was applied to both metabolites (rows) and individual samples (columns) to identify patterns of metabolite variation. The color gradient represents the standardized metabolite abundance, with red indicating upregulation and blue indicating downregulation.

The separation of the treatment groups by PCA was driven by 21 serum metabolite concentrations that differed between NCG and CON groups. The hierarchical clustering heatmap (Figure 3) identified 40 metabolites that revealed two distinct sub-clusters within the NCG ewes, driven by characteristic metabolic patterns. Specifically, one subgroup of NCG ewes displayed a marked depletion of branched-chain amino acids (valine, leucine, isoleucine), glycine, proline, and phosphate-metabolites that were relatively abundant in the CON ewes. Conversely, the second NCG cluster showed higher levels of metabolites such as serine, ethanolamine, urea, and 2-hydroxyhexanoic acid. From the 21 serum metabolite concentrations that differed between NCG and CON groups, 11 had *p* < 0.05 and FDR < 0.05, where 6 metabolites decreased (phosphate, glycine, proline, isoleucine, valine, leucine) and 5 increased (methanolphosphate, 2-hydroxypyridine, serine, ethanolamine, 2-hydroxyhexanoic acid) in the NCG compared to CON group (Table 4).

#### 3.5.2. Pathway Topology Analysis

**Figure 4:**
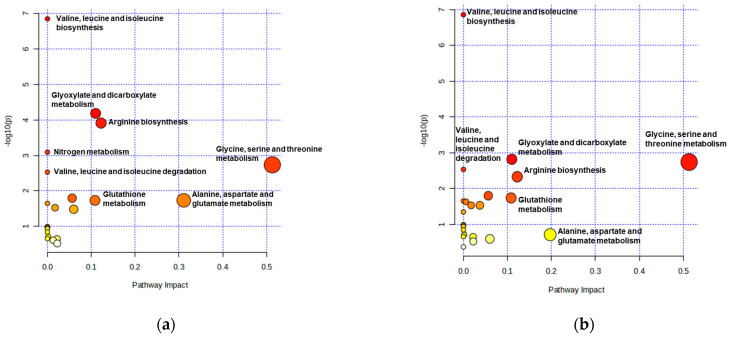
Summary of Pathway Analysis, considering all metabolites (**a**) and metabolites with significantly different levels (*p* ≤ 0.05) (**b**). The x-axis represents pathway enrichment, and the y-axis represents pathway impact. In both panels, circle size represents the pathway impact, and the color gradient (from yellow to red) indicates the level of statistical significance (red = higher significance).

Pathway topology analysis revealed that NCG supplementation affected several key metabolic routes, including valine, leucine and isoleucine biosynthesis and degradation, glyoxylate and dicarboxylate metabolism, arginine biosynthesis, nitrogen metabolism, glutathione metabolism, glycine, serine and threonine metabolism, and alanine, aspartate and glutamate metabolism (Figure 4a). These alterations were associated with reduced concentrations of branched-chain amino acids (valine, leucine, isoleucine), glycine, proline, and phosphate, together with increased levels of serine, ethanolamine, urea, and 2-hydroxyhexanoic acid. When only significantly altered metabolites (*p* ≤ 0.05, Mann–Whitney test) were considered, additional pathways such as pyrimidine metabolism, phenylalanine metabolism, glycerolipid metabolism, and arachidonic acid metabolism were identified (Figure 4b). Overall, the observed metabolite shifts indicate changes in amino acid, nitrogen, and phospholipid metabolism, consistent with stimulation of the urea cycle and reorientation of maternal nutrient utilization during late gestation.

## 4. Discussion

Improving fetal growth and colostrum quality is a critical strategy to reduce neonatal lamb mortality, particularly in undernourished ewes [10]. The key findings of this study were that NCG supplementation during the last third of gestation to twin-bearing ewes under nutrient restriction (~50% NRC requirements), despite generating blood metabolite changes in the ewe, had no effect on fetal weight at term. However, NCG supplementation was associated with an increase in protein and IgG content in the colostrum. Thus, NCG supplementation in pregnant ewes in nutrient-restricted environments may potentially contribute to improved lamb survival through improved colostrum quality.

In the present study, maternal body weight and BCS were not influenced by NCG supplementation. This finding suggests that in twin-bearing ewes under a 50% nutrient restriction, NCG did not contribute to additional maternal tissue accretion. The absence of an effect may be related to the limited nutrient supply available to support maternal growth, regardless of the potential stimulatory role of NCG in nitrogen metabolism. Our results align with the idea showing that, when energy and protein are constrained, supplementation with NCG alone is insufficient to alter maternal weight trajectories during gestation. While previous studies have reported similar outcomes with NCG supplementation from day 35 to 110 of gestation [33] or from 100 to 140 dg [31] or arginine from day 100 to 125 of gestation [40], or from 60 to parturition [16], or in 60% nutrient-restricted ewes supplemented with rumen-protected arginine at doses of 180 mg/kg BW during last two-thirds of gestation [41], the current findings strengthen this evidence by demonstrating that even with NCG supplementation, maternal body reserves remain primarily dependent on overall nutrient availability.

In the present study, daily NCG supplementation from day 100 of gestation until lambing did not affect lamb birth weight. This result suggests that, under conditions of maternal undernutrition and elevated nutrient requirements of twin-bearing ewes, NCG was unable to counteract growth restriction. One explanation may be the timing of supplementation. Since treatment began after placentation was completed [42], no effect on placental weight was observed, limiting the potential for improved nutrient transfer to the fetus as the placenta plays a major role in fetal growth [43]. This notion is supported by previous studies where undernourished (50% NRC) twin-bearing ewes supplemented with similar doses of NCG before the end of placentation augmented the placentome weight [44], likely enhancing nutrient transfer, resulting in an 18% higher fetal body weight compared to unsupplemented animals [29,45]. Another explanation may relate to the high nutritional demands of twin pregnancies, where limited nutrient availability likely constrained the capacity of NCG to enhance fetal growth, consistent with our prior study where ewes were even more severely undernourished (30% of NRC requirements) [31]. In contrast, a study in well-fed cattle in the last 28 dg supplemented with NCG increased calf weight [46] suggesting that meeting the nutrient requirements of pregnant ruminants may be required for NCG to enhance fetal growth. Interestingly, previous studies have reported improved fetal growth following Arg supplementation to undernourished ewes in a timeframe similar to the present study [16,17]. The reason for the differences between studies is unclear but may be related to the availability of amino acids to support endogenous Arg synthesis via the urea cycle in the present study. Taken together, our results emphasize that maternal nutrient supply is a critical determinant of fetal outcomes and that supplementation initiated late in gestation is insufficient to improve birth weight under restricted conditions.

Supplementation with NCG resulted in an increase in colostrum protein and IgG content compared to CON ewes. Although colostrum yield was not measured and the impact on lamb colostrum intake and immune transfer remains to be evaluated, the observed compositional improvements indicate that NCG may enhance neonatal nutritional and immunological support. This could be beneficial for lamb survival, as the survival of neonates relies entirely on the colostrum intake [47], its nutrients [48], and immunoglobulin (IgG) to achieve immunological protection [47]. The increase in protein content is consistent with previous reports in dairy cows, where NCG supplementation during late gestation and lactation [49,50,51], likely acts through enhanced amino acid supply, mammary plasma flow, and amino acid utilization in the mammary gland [49]. Additionally, the increase in IgG content is consistent with earlier studies showing improved immunological status following NCG supplementation in finishing Holstein bulls [51] and lactating cows under stress [51]. Although the precise biological mechanisms by which NCG increases colostrum IgG remain unclear, it is plausible that, similar to Arg, NCG supports immune function through effects on B lymphocyte differentiation and immunoglobulin secretion [52], ultimately improving colostrum quality.

To our knowledge, this study is the first to evaluate the effect of NCG supplementation during the last third of gestation on nutrient-restricted twin-bearing ewes’ metabolome. Although an explained variance above 70–90% in the first two principal components is often considered desirable in multivariate analyses [53], we acknowledge that our PCA did not reach this threshold. This is a common limitation in metabolomics, where the high dimensionality and biological complexity of the data frequently result in lower percentages explained by PC1 and PC2 [54,55,56]. In our dataset, these two components together explained 59.2% of the variance, which falls within the range often reported in metabolomics studies (typically 40–60% for the first two PCs). Importantly, when including the first five components, the cumulative variance explained reached 80.5%, which we believe supports the adequacy of the model. Taken together, this cumulative variance provides a reasonable representation of the metabolomic differences observed between NCG and CON groups, while also highlighting the inherent complexity of untargeted metabolomics data.

Both conventional blood biochemical evaluations and untargeted metabolomics, revealed shifts in metabolic pathways, particularly those related to the urea, amino acid, energy, ammonia recycling, arginine and proline, phospholipid biosynthesis, and fatty acid metabolism. NCG activates [24] and improves [50] the urea cycle, with greater conversion of ammonia to urea. Interestingly, although blood urea concentration in pregnant ewes was not affected [33], other studies in cattle and supplementation timings indicate that NCG supplementation reduces urea in blood [24]. In contrast with these findings, in the present study, although the blood serum levels of urea were within the normal range for sheep, NCG-supplemented animals had higher blood urea nitrogen concentration compared to CON animals. The main difference with the Zhang et al. [33] study in pregnant ewes was the timing of NCG supplementation. While this study was done from day 35 to 110 of gestation, in the present study, animals were under a higher nutrient demand in mid- to late-gestation [6]. This high nutrient demand was reflected by the depletion of specific amino acids in maternal blood. Although no differences were observed between NCG and CON animals, when observing the normal range values for the specie (see Table 4), maternal nutrient restriction resulted in plasma metabolite changes. Both groups presented increased β-hydroxybutyrate with values over the limit for subclinical ketosis (>0.8 mmol/L) [57]. In addition, total protein content and serum albumin concentration were reduced in both groups, suggesting a metabolic compromise of the animals, with a deficient nutrient intake and increased lipid reserve catabolism to sustain nutrient requirements during late gestation. Therefore, regardless of the limited nutrient intake and increased nutrient demands with advancing gestation, NCG-supplemented ewes still reflected an effect on urea metabolism. This may be indicative of a higher ammonia conversion into urea.

Metabolic pathway analysis identified arginine, valine, leucine and isoleucine biosynthesis and nitrogen metabolism as key metabolic pathways affected by NCG supplementation. Although Arg was not detected in the metabolomic analysis, metabolites involved in the Arg biosynthesis pathway appear affected by NCG supplementation. However, the level of impact, based on the number of metabolites involved in the pathway, was reduced (Supplementary Table S1), contrasting with the role of NCG in endogenous Arg synthesis. These results could be explained by the prioritization of ammonia detoxification, via increased urea synthesis. Other effects observed in NCG animals were the reduced concentration of glucogenic amino acids glycine and valine, which serve as energy source by the fetus [58], similarly to leucine and valine [59] which play an important role in fetal growth and placental function. These results may reflect partitioning of nutrients towards the fetus, leading to reduced maternal plasma concentrations. However increased uptake of branched-chain amino acids by placenta cannot be excluded [60], particularly given the absence of an effect on fetal growth. Nevertheless, as there was no change in the placental or fetal traits measured, the function of these metabolic changes remains to be elucidated.

A severe decrease in phosphate was observed in NCG-supplemented animals compared to CON animals. This effect may be associated with metabolic changes, as phosphate plays a major role in maintaining normal physiological functions [61]. However, the specific significance of decreased phosphate levels in the blood of NCG-supplemented animals is unclear, as both groups received the same nutrition and there was no significant maternal or fetal tissue accretion in the NCG group compared to CON animals. The decrease in blood glycine in NCG-supplemented ewes compared to controls correlates with an increase in serine concentrations. Both glycine and serine are non-essential amino acids that share a common pathway and can interconvert [62]. Serine plays a crucial role in various metabolic pathways, including energy production, and its increase may reflect prioritization toward gluconeogenesis [62], a response to oxidative stress [63] and/or ammonia recycling [62]. Altogether, these data reinforce the notion that the animals were experiencing nutrient and energy stress. This is further supported by the increase in 2-hydroxyhexanoic acid, which was more abundant in NCG-supplemented compared to CON ewes. 2-Hydroxyhexanoic acid is a branched-chain alpha-keto acid associated with branched-chain amino acid metabolism (leucine, isoleucine, and valine) [64,65] and lipid metabolism [66]. When combined with the observed changes in amino acid profiles, these findings suggest a metabolic shift toward fatty acid oxidation to support energy production under nutrient restriction.

Colostrum of NCG-supplemented ewes had higher protein and IgG concentrations compared to CON ewes. In the present study, NCG animals had reduced concentrations of the branched-chain amino acids leucine, isoleucine, and valine, which are essential amino acids that regulate various physiological processes during pregnancy in sheep. Reduced maternal plasma concentrations of these amino acids could be associated with their uptake by the mammary gland for colostrum protein formation, as these amino acids have important roles in mammary protein synthesis [67], comprising 50% of the essential amino acids in milk proteins [68], and also contributing to the immune system [69]. Nevertheless, their mechanisms of action have not been clearly defined. In addition to its glucogenic role, the reduced glycine in NCG-supplemented ewes may also reflect higher uptake for protein production, potentially in the colostrum, as it is rich in this and other amino acids [70], and may contribute to the increase in protein concentration in the colostrum of NCG ewes. Therefore, our results suggest that NCG supplementation influenced some of the serum metabolites, which may reflect the increased utilization of specific metabolites (e.g., proline, valine, isoleucine, and leucine) for colostrum production. However, the biological function of these changes, in the absence of phenotypic shifts (e.g., live weight, body condition, and fetal growth and immunological function), remains unclear and warrants further investigation.

A final consideration relates to body condition score (BCS), which is a critical indicator not only of nutritional status but also of animal welfare. Maintaining ewes within an optimal BCS range is essential to support both maternal health and successful reproduction. Undernutrition during pregnancy, as modeled in this study, reduces BCS and may compromise ewe resilience, metabolic balance, and overall welfare. While our findings show that NCG supplementation did not influence BCS, this parameter must be carefully monitored in research and production settings to avoid adverse effects on maternal well-being. Future interventions aimed at improving fetal growth or colostrum quality, such as stocking rate adjustment, supplementation, improved nutrient management, adjustment of the breeding season, among others, should therefore be considered to preserve or enhance ewe BCS, ensuring alignment with welfare standards alongside productivity goals.

## 5. Conclusions

This study demonstrates that 60 mg/kg BW NCG supplementation in undernourished twin-bearing ewes, from day 100 of gestation until lambing, does not improve fetal growth. However, NCG supplementation improved colostrum composition by increasing both total protein and IgG concentrations, which are essential for neonatal survival. Metabolomic and biochemical blood serum analyses suggest that NCG enhances, at least partially, nitrogen metabolism and urea cycle efficiency in twin-bearing ewes, even under conditions of restricted maternal nutrition. The observed alterations in amino acid profiles, particularly reductions in glucogenic and branched-chain amino acids, may reflect increased mammary gland uptake to support colostrogenesis. These findings highlight the potential of NCG to improve colostrum quality, although further research is needed to determine optimal timing, dosage, and the mechanisms underlying its effects on colostrum composition and neonatal outcomes, as well as the potential role of NCG in animals under less restricted nutrient conditions.

## Figures and Tables

**Table 1:** Birth weight, body dimensions and rectal temperature 30 min post-partum, gestation length of lambs and fetal membrane and cotyledon data from undernourished twin-bearing ewes supplemented with NCG compared to unsupplemented controls (CON) ^1^.

	CON	NCG	*p*-Value		
			*Treatment*	*Sex*	*T × S*
** Body Weight (kg) and Dimensions (cm) **					
Body weight	3.05 ± 0.10	2.95 ± 0.09	0.45	0.13	0.94
Body length	35.22 ± 0.63	36.21 ± 0.62	0.26	0.40	0.72
Biparietal diameter	6.04 ± 0.06	6.08 ± 0.06	0.65	0.12	0.11
Thorax diameter	34.01 ± 0.49	36.62 ± 0.48	0.58	0.91	0.78
Front leg length	30.23 ± 0.35	29.53 ± 0.34	0.16	0.02	0.07
Hind leg length	35.50 ± 0.46	35.57 ± 0.46	0.91	0.04	0.49
** Other newborn traits **					
Gestation length (days)	143.90 ± 0.84	143.50 ± 0.84	0.74	0.97	0.98
Lamb rectal temperature (°C)	36.67 ± 0.78	38.21 ± 0.75	0.12	0.17	0.22
** Fetal membranes and cotyledons **					
Total p weight (g)	554.50 ± 18.95	536.00 ± 18.47	0.49		
Total cotyledon weight (g)	208.10 ± 8.35	181.8 ± 7.92	0.16		
Total number of cotyledons	90.10 ± 2.09	85.40 ± 2.04	0.12		

^1^ Data are presented as means ± SEM. *p*-value for treatment, sex of the fetus (*sex*) and treatment by sex interaction (*T* × *S*) are presented.

**Table 2:** Maternal blood metabolites at 130, 140 days of gestation (dg) and post lambing, from undernourished twin-bearing ewes supplemented with NCG compared to unsupplemented controls (CON) ^1^. Normal range for each metabolite in sheep plasma is presented [39].

Metabolite	Days				*p*-Value		Normal Range
	130 dg	145 dg	Post Lambing	Group	Time	Group × Time	
**β-hydroxybutyrate (mmol/L)**							
CON	1.06 ± 0.05	1.09 ± 0.06	0.87 ± 0.05	0.99	0.01	0.07	0.2–0.6
NCG	1.04 ± 0.05	0.99 ± 0.06	0.97 ± 0.05				
**Total protein (g/L)**							
CON	61.00 ± 1.57	49.62 ± 1.66	57.42 ± 1.6	0.57	<0.001	0.90	68–88
NCG	62.05 ± 1.57	51.20 ± 1.66	58.03 ± 1.6				
**Albumin (g/L)**							
CON	25.45 ± 0.60	23.64 ± 0.64	24.20 ± 0.61	0.87	<0.001	0.16	26–42
NCG	26.50 ± 0.60	23.01 ± 0.64	23.95 ± 0.61				
**Urea (mmol/L)**							
CON	4.21 ± 0.21	4.59 ± 0.24	4.19 ± 0.22	0.03	0.01	0.50	4.0–10.0
NCG	4.73 ± 0.21	5.30 ± 0.24	4.44 ± 0.22				

^1^ Data are presented as means ± SEM.

**Table 3:** Colostrum composition from orally supplemented with NCG and control (CON) undernourished ewes, obtained 15–30 min after the second lamb was delivered, from both sides of the udder, before the suckling of the lambs ^1^.

	CON	NCG	*p*-Value
Fat (%)	13.67 ± 0.89	14.99 ± 0.89	0.30
Non-fat solids (%)	26.11 ± 1.66	29.84 ± 1.66	0.12
Density (g/cm^3^)	1.08 ± 0.06	1.09 ± 0.06	0.23
Protein (%)	13.59 ± 0.94	16.45 ± 0.94	0.04
IgG (mg/mL)	57.48 ± 2.82	66.49 ± 2.89	0.03

^1^ Data are presented as means ± SEM.

**Table 4:** Concentration change (arbitrary units, mean ± SEM), percent difference, *p*-value, false discovery rate (FDR), fold change (CON/NCG) and log2(FC) of serum metabolites with a significant *p*-value and FDR, in serum samples from undernourished twin-bearing ewes treated (NCG) or not (CON) with N-carbamylglutamate.

Metabolite	CON	NCG	Percent Difference	*p*-Value	FDR	Fold Change	log2(FC)
Phosphate	52.27 ± 33.12	0.01 ± 0.001	−99.98	<0.001	0.0085	−5227	−12.5
Glycine	31.17 ± 16.27	0.03 ± 0.01	−99.90	<0.001	0.0010	−706	−10.1
Proline	0.63 ± 0.26	0.02 ± 0.01	−96.83	<0.001	0.0010	−32	−5.0
Isoleucine	2.03 ± 1.30	0.14 ± 0.07	−93.10	0.002	0.0284	−15	−3.9
Valine	1.04 ± 0.57	0.08 ± 0.03	−92.31	<0.001	0.0010	−13	−3.7
Leucine	2.53 ± 1.24	0.43 ± 0.09	−83.00	<0.001	0.0046	−28	−2.5
Methanolphosphate	0.07 ± 0.19	0.51 ± 0.12	628.57	<0.001	0.0010	2	2.8
2-hydroxypyridine	0.09 ± 0.16	0.41 ± 0.10	355.56	<0.001	0.0072	1	2.1
Serine	6.63 ± 6.06	30.56 ± 13.78	360.94	<0.001	0.0072	2	2.2
Ethanolamine	10.43 ± 29.28	68.62 ± 14.89	557.91	<0.001	0.0010	1	2.7
2-hydroxyhexanoic acid	0.96 ± 0.43	69.44 ± 10.66	7133.33	<0.001	1.52 × 10^−11^	11	6.2

## Data Availability

Data is contained within the article.

## Supplementary Materials

The following supporting information can be downloaded at https://www.mdpi.com/article/10.3390/ani15202998/s1: Table S1: Significantly altered metabolic pathways of metabolites obtained from undernourished twin-bearing ewes treated (NCG) or not (CON) with N-carbamylglutamate; Table S2: The first five principal components and the loading scores of the top contributing metabolites for PC1 to PC5.

## Abbreviations

The following abbreviations are used in this manuscript:

NCG	N-carbamylglutamate
BW	Body weight
NRC	National Research Council
Arg	Arginine
dg	Days gestation
BCS	Body Condition Score
