# Supplementary material for: Effect of N-Carbamylglutamate Supplementation in Late Pregnancy on Nutrient-Restricted Twin-Bearing Ewes on the Pre-Lambing Maternal Metabolome, Colostrum Quality and Lamb Birth Weight
Source: Animals (Basel). 2025 Oct 16;15(20):2998. doi: 10.3390/ani15202998 (PMC12560894; doi:10.3390/ani15202998)
Supplement: Supplementary file 1 [file animals-15-02998-s001.zip › Supplementary_Material 1.pdf]

Supplementary Material

1 Supplementary Table

Supplementary Table S1. Significantly altered metabolic pathways of metabolites obtained from undernourished twin-bearing ewes treated (NCG) or not (CON) with N-carbamylglutamate.

Pathways	Total	Hits	-LOG10(p)	FDR	Impact
Glycine, serine and threonine metabolism	34	3	2.74	0.05	0.51
Alanine, aspartate and glutamate metabolism	28	1	0.71	0.87	0.2
Arginine biosynthesis	14	2	2.33	0.08	0.12
Glyoxylate and dicarboxylate metabolism	32	3	2.81	0.05	0.11
Glutathione metabolism	28	2	1.73	0.21	0.11
Pyrimidine metabolism	38	1	0.59	0.98	0.06
One carbon pool by folate	26	2	1.8	0.21	0.06
Glycerophospholipid metabolism	36	2	1.53	0.22	0.04
Primary bile acid biosynthesis	46	1	0.52	1.00	0.02
Cysteine and methionine metabolism	33	1	0.64	0.91	0.02
Arginine and proline metabolism	36	2	1.53	0.22	0.02
Sphingolipid metabolism	32	2	1.62	0.21	0.01
Lipoic acid metabolism	28	1	0.71	0.87	0
Valine, leucine and isoleucine biosynthesis	8	4	6.85	1.12E-05	0
Nitrogen metabolism	6	1	1.34	0.30	0
Valine, leucine and isoleucine degradation	40	3	2.53	0.06	0
D-Amino acid metabolism	14	1	0.99	0.62	0
Butanoate metabolism	15	1	0.96	0.62	0
Porphyrin metabolism	31	2	1.65	0.21	0
Histidine metabolism	16	1	0.93	0.62	0
Pantothenate and CoA biosynthesis	20	1	0.84	0.72	0
Glycosylphosphatidylinositol (GPI)-anchor biosynthesis	32	1	0.65	0.91	0
Purine metabolism	71	1	0.37	1.00	0
